# Evaluation of the effect of *Echium amoenum* on pain control after clinical crown lengthening surgery

**DOI:** 10.34172/japid.2023.003

**Published:** 2023-05-03

**Authors:** Shima Ghasemi, Fatemeh Bakhtiari, Pariya Rahimi Asl, Amirreza Babaloo, Atieh Ghasemi

**Affiliations:** ^1^Department of Prosthodontics, Faculty of Dentistry, Tabriz University of Medical Science, Tabriz, Iran; ^2^Student Research Committee, Faculty of Dentistry, Tabriz University of Medical Science, Tabriz, Iran; ^3^Department of Periodontics, Faculty of Dentistry, Tabriz University of Medical Science, Tabriz, Iran; ^4^Department of Pediatrics, Faculty of Medicine, Tabriz University of Medical Science, Tabriz, Iran

**Keywords:** Clinical tooth crown lengthening surgery, Echium amoenum, Pain control, Periodontal surgery

## Abstract

**Background.:**

Pain after periodontal surgeries is one of the most common complications. *Echium amoenum* is among the important therapeutic herbs in Iranian traditional medicine. Various studies have shown its pain control properties. This study aimed to evaluate this herb’s efficacy in controlling pain after periodontal surgeries.

**Methods.:**

In this randomized clinical trial, 50 patients referred to Tabriz Dental School for clinical crown lengthening surgery were divided into two equal groups: control and test. In the test group (using *E. amoenum*), 24 hours before surgery, *E. amoenum* was administered to the patient at home every 12 hours, and a dose of *E. amoenum* was administered one hour before the procedure. The postoperative pain was assessed using VAS 30 minutes, 1 hour, and 3 hours after the surgery and verbal rating scale (VRS) 24, 48, and 72 hours postoperatively. A chi-square test was used to compare the pain severity between the two groups.

**Results.:**

The VAS index was significantly lower in the *E. amoenum* group compared to the control group (30 minutes, 1 hour, and 3 hours after the procedure) (*P*<0.05). In both groups, pain severity increased significantly up to 3 hours postoperatively (*P*<0.05). In the first 24 hours, the VRS index of the *E. amoenum* group was significantly lower than that of the control group (*P*<0.05), with no significant differences between the two groups at 48 and 72 hours (*P*>0.05).

**Conclusion.:**

The *E. amoenum* herb reduced the pain severity after clinical crown lengthening surgeries.

## Introduction

 Pain after periodontal surgeries is among the most common unpleasant problems and complications for patients.^1,2^ After surgery, inflammation occurs in the periodontal tissues due to tissue trauma, and the subsequent restoration and tissue damage will damage the cell wall. With the help of phospholipase, it will lead to cell membrane rupture and the release of membrane phospholipids, which in turn will be transformed into prostaglandins, thromboxane, and other metabolites by COX-1 and COX-2 enzymes, causing pain.^3,4^ However, many factors, such as the patient’s temperament, the duration, type, and extent of the surgery, psychological aspects such as stress and excitement, the extent of the tissue trauma, duration of the gingival disease, and type of the anesthetic agent used (long-acting, moderate-acting, or short-acting) may affect the pain severity, making the accurate comparison of the studies in this area difficult.^1,2^ Traditional medicine, as an inspiration for human health, has played a major role in replacing chemical medicines with various herbal medicines in some countries, such as Iran. Moreover, the tendency of the Iranian society to use traditional herbal treatments due to their harmlessness and cost-effectiveness has led to the widespread use of herbal medicines in traditional medicine.^5^

*Echium amoenum* is one of the important therapeutic herbs in Iranian traditional medicine.^6^ This plant has been used as a demulcent, anti-inflammatory and analgesic, anxiolytic, and sedative in the folk medicine of Iran. Several studies have evaluated this herb in recent years, investigating its antibacterial effects,^7^ the essential oil components of petals,^8^ pyrrolizidine alkaloids,^9^ phenolic compounds,^10^ and anxiolytic,^11^ analgesic,^12^ and anticonvulsive effects.^13^ In addition, its antioxidant effects,^14^ mild-to-moderate anti-depressive effects,^15^ and its effect on mental disorders have been studied.^16^ All of these studies have been conducted by Iranian researchers on the herbal organ of the *E. amoenum* flowers. A study by Abed et al^17^ showed that *E. amoenum* significantly decreased pain and inflammation in acute pancreatitis patients. However, based on a literature search, no study has investigated the effect of this herb on pain control after gingival surgeries. Hence, this study was conducted to determine whether *E. amoenum* can control pain after periodontal surgeries and whether *E. amoenum* can replace or at least supplement conventional medications used after periodontal surgeries.

## Methods

 In this randomized clinical trial with a placebo group and parallel, double-blind groups, random sampling was performed among the people referring to the Periodontics Department of the Faculty of Dentistry, Tabriz University of Medical Sciences, for clinical crown lengthening. The study was conducted according to the declaration of Helsinki.

 Based on a study by Babaloo et al^18^ and considering the alpha value of 0.05 and a test power of 80%, the sample size was determined at n = 21 in each group. To increase the study’s validity, 20% was added to this number, and 25 samples were included in each group.

 Fifty patients were selected after meeting the research inclusion criteria and randomly assigned to the control or examined group using the randomizer website at randomizer.org. The research inclusion criteria included informed consent of the patients, patients undergoing dental crown lengthening (in the posterior maxilla) surgery, an age range of 20‒65 years, and patients’ physical and mental health. The research exclusion criteria also included patients with a systemic or mental illness, consuming alcohol or any psychotropic drugs, known sensitivity to NSAIDs, caffeine, or acetaminophen, any bleeding disease, patients at risk of any infection after surgery, patients with hepatic problems, patients with heart problems and hypertension, patients who did not have the necessary cooperation during the study, patients with mental and physical disabilities (patients without qualification to give personal consent and complete the questionnaires), patients in which the use of *E. amoenum* was harmful, patients who needed increased doses of analgesic drugs, patients with no need for bone surgery, and pregnant and lactating women. The necessary explanations were provided to the patients, and their written informed consent was obtained. The caregivers of the patients who could not read and sign the informed consent form (for example, illiterate subjects) were asked to assist them in these steps. One periodontist performed the clinical crown lengthening surgery, who was blinded to the procedures.

 Before the surgical phase, the patient was motivated and given oral hygiene instructions for the nonsurgical removal of calculus. The surgery site in all the patients was in the posterior maxilla, and the bone removal site was about 3‒4 mm between the bone margin and the healthy edge of the tooth. The surgery duration was the same for all the patients (approximately 20 minutes). Topical infiltration technique and 2% lidocaine with 1:800 000 epinephrine were used for anesthesia (one cartridge was used in each area). A full-thickness flap was elevated using sulcular incisions of one tooth beyond the surgical site without a releasing flap. After surgery, the area was closed with a vertical mattress suture. After surgery, the patients were advised not to spit, not to drink through a straw, to avoid chewing in the areas of surgery, to avoid hard food, to drink plenty of liquids, not to use alcohol, and not to smoke.

*Echium amoenum* was used in the first group. *E. amoenum* was monitored and consulted with the Faculty of Pharmacy of Tabriz University of Medical Sciences. Each pack was prepared with a demo bag from the Golestan brand in 250 mL of water in a covered container. Twenty-four hours before the surgery and every 12 hours, a dose of *E. amoenum* was used by the patients at home. The second group used the placebo (conventional drinking water in a covered container).

 Due to ethical considerations, after the surgery, the patients in both groups were given an analgesic drug (Novafen) according to the surgeon’s guidance. The first dose of the medicine was administered immediately after the end of the surgery, and the subsequent doses were used at 6-hour intervals after the first dose for three days.

 The level of pain after the surgery was assessed 30 minutes, 1 hour, and 3 hours after the surgery using the visual analog scale (VAS), completed by the patients at home. The pain level was assessed 24 hours, 48 hours, and 72 hours after the surgery using the verbal rating scale (VRS), completed and evaluated through phone contact with the patients. In this measurement scale, the rating system used was as follows: 0: (pain-free), 1: mild pain (tolerable), 2: moderate pain (significant pain relieved by the administrated analgesic drug), 3: severe pain (significant pain that was not relieved by the administrated analgesic drug), and 4: very severe pain. No antibiotic was administrated to the patients.

 Friedman test was used to assess the pain severity in two groups 30 minutes, 1 hour, and 3 hours after the surgery using the VAS index, and the pain severity in the two groups 1, 2, and 3 days after the surgery was assessed using the VRS index. A chi-square test was used to compare the pain severity between the two groups. Statistical analysis was performed using SPSS 17, and *P*< 0.05 was considered significant.

## Results

 The results showed the highest pain in the control group 3 hours after the surgery (6.44 ± 1.38), with the lowest pain in the test group 30 minutes (0.72 ± 0.54) and 24 hours (0.72 ± 0.52) after the surgery.

 According to the results, there were significant differences between the control and test groups regarding pain severity. To examine the significance of this difference, inferential statistics were used. In addition, since the data were of ranked type data, Friedman and Wilcoxon’s tests were used for data analysis.

###  VAS

 The Wilcoxon test was used to compare pain in the control and experimental groups at the study intervals. The results revealed that the statistical value of the test used to assess the pain severity in the control and experimental groups was significant at all three intervals (30 minutes, 1 hour, and 3 hours) (P = 0.00). Therefore, the patients’ pain in the control and test groups differed significantly at all the three intervals. In addition, the Wilcoxon test showed that the pain severity in the test group was significantly lower than that of the control group at 30 minutes, 1 hour, and 3 hours postoperatively.

 Friedman test was used to compare patients’ pain in the control group. There was a significant difference in patients’ pain in the control group at study intervals (*P*= 0.00). The Wilcoxon test showed that pain was significantly different at all three intervals (*P*= 0.05). According to the results of the Wilcoxon test, pain severity in the control group increased significantly up to 3 hours after the surgery (*P*= 0.00). The test group patients at the study intervals exhibited the same results as the control group (*P*< 0.05).

###  VRS

 The Wilcoxon test was used to compare pain severity in the control and test groups at the study intervals. There was a significant difference between the test statistic values related to the patients’ pain in the control and test groups 24 hours postoperatively (*P*= 00.00). However, 48 hours (*P*= 00.26) and 72 hours (*P*= 00.674) after the surgery, no significant difference was found between the patients of the control and test groups. Therefore, pain severity in the control group increased significantly 24 hours after the surgery, with no significant differences between the pain scores of the control and test groups 48 hours and 72 hours postoperatively.

 The Friedman test was used to compare pain in the control group at the study intervals. There was a significant difference in pain severity in the control group patients between the three intervals (*P*= 0.00). According to the Wilcoxon test, there was a statistically significant difference in pain scores between the three intervals in the control group (*P*< 0.05). Therefore, pain severity in the control group decreased significantly from 24 hours after surgery to 72 hours after surgery (*P*< 0.05).

 The Friedman test was used to compare pain in the control group at the study intervals. There were no significant differences in the pain scores in the test group at 24, 48, and 72 hours postoperatively (*P*= 0.794).

## Discussion

 In most surgeries, the tissue is damaged, resulting in varying degrees of inflammatory response and pain. Postoperative guidelines and care are important factors leading to successful treatment.^19^

 Fifty patients referring to the Department of Periodontics, Tabriz University of Medical Sciences, for crown lengthening, were included in the study to evaluate the effect of the *Echium amoenum* on postoperative pain control.

 The pain severity depends on several factors such as the type of dental treatment (surgical, endodontic, periodontal), the type of periodontal surgery, the patient’s mental condition, gender, duration of surgery, level of the trauma, duration of the gingival disease, etc.^1,2^ Thus, this study sought to assign the patients to two homogeneous groups as far as possible.

 Based on the present study, the pain severity was significantly lower in the *E. amoenum* group compared to the other group. At the same time, there were no significant differences between the two groups at 30-minute and one-hour postoperative intervals due to the anesthetic effect. However, the pain severity increased significantly up to 3 hours after the surgery in both groups. Heidari et al^12^ examined the analgesic effects of *E. amoenum* on mice. The results showed that *E. amoenum* extract had good analgesic effects. In the present study, the VRS index indicated the pain severity 1, 2, and 3 days after the surgery, with a significant difference between the two groups on the first day. The anti-inflammatory properties of *E. amoenum* were responsible for pain relief,^12^ consistent with the current study.

 Saponin is among the ingredients of *E. amoenum* that improves memory and reduces anxiety.^20^ Given the anxiolytic property of *E. amoenum*, the group receiving *E. amoenum* experienced less pain even in the early hours of the day due to reduced anxiety and higher mental relaxation. Sayyah et al^21^ found that *E. amoenum* had an analgesic effect on patients with generalized anxiety disorder. Faryadyan et al^22^ showed that the hydroalcoholic extract of *E. amoenum* could have anxiolytic effects by increasing dopamine levels. By modulating the function of serotonergic nervous systems that play a key role in reducing anxiety, depression, and neurological disorders, it reduces the level of anxiety. In addition, no significant difference was found between the two groups two and three days after the study. Due to reduced pain severity two and three days after the study, the pain severity was similar in the two groups.

 Moreover, the sedative effect of *E. amoenum* was another cause of lower pain in the patients using the *E. amoenum*. A study by Abed et al^17^ also showed that *E. amoenum* significantly reduced pain and inflammation in acute pancreatitis patient.

## Conclusion

 Based on the results of the present study, *E. amoenum* reduced the pain severity after clinical crown lengthening procedures. The pain severity decreased two days after the clinical crown lengthening surgery in normal conditions; therefore, the effect of this herb decreased.

## Acknowledgments

 Not applicable.

## Availability of Data

 The datasets used and/or analyzed during the current study are available from the corresponding author on reasonable request.

## Competing Interests

 All authors declare that they have no conflicts of interest.

## Ethical Approval

 The protocol of the study was approved by the Ethics Committee of Tabriz University of Medical Sciences under the code IR.TBZMED.REC.1398.373. In addition, the study was registered at the Clinical Trials Website of the Ministry of Medical Education (IRCT) under the code IRCT20180630040290N3.

## Funding

 Not applicable.
